# Aeromonas hydrophila: A Rare Cause of Deep Visceral Abscess

**DOI:** 10.7759/cureus.81522

**Published:** 2025-03-31

**Authors:** Mouad Harandou, Abderrazak Saddari, Said Ezrari, Mohamad Lahmer, Jalal Abderrahmani, Elmostafa Benaissa, Yassine Ben Lahlou, Mostapha Elouennass, Adil Maleb

**Affiliations:** 1 Microbiology, Faculty of Medicine and Pharmacy, Mohammed VI University Hospital, Mohammed First University, Oujda, MAR; 2 Bioresources, Biotechnology, Ethnopharmacology and Health, Faculty of Sciences, Mohammed VI University Hospital, Mohammed First University, Oujda, MAR; 3 Bacteriology, Mohammed V Military Training Hospital, Rabat, MAR; 4 Microbiology, Faculty of Medicine and Pharmacy, Mohammed V University, Rabat, MAR

**Keywords:** aeromonas hydrophila, aeromonas infection, antibiotherapy, deep visceral abscess, pancreatic abscess

## Abstract

*Aeromonas hydrophila* is a rare but notable pathogen associated with deep visceral abscesses, especially in patients with underlying malignancies or immunosuppression. Commonly found in aquatic environments, it is typically implicated in gastrointestinal infections but can also cause severe conditions such as sepsis or necrotizing fasciitis. In this report, we describe the case of a 76-year-old female patient diagnosed with pancreatic adenocarcinoma and a concurrent abscess in the pancreatic region caused by *A. hydrophila*. The patient presented with epigastric pain, jaundice, and elevated liver biomarkers. After receiving systemic antibiotic therapy with gentamicin and ceftriaxone, the patient achieved full recovery. This case highlights the importance of recognizing *A. hydrophila* as a rare cause of deep visceral abscesses, emphasizing the need for early diagnosis and effective management to improve prognosis and survival rates.

## Introduction

*Aeromonas* spp. are primarily zoonotic microorganisms that have recently gained recognition as emerging human pathogens. Predominantly thriving in aquatic environments, these bacteria pose significant concerns due to their intrinsic resistance to multiple antibiotics [1]. Aeromonas infections of the hepatobiliary system, including hepatic and pancreatic abscesses, have a reported mortality rate of up to 11%, particularly in patients with underlying comorbidities [2]. In the hepatobiliary tract, they are most commonly implicated in cholangitis, with only rare cases of deep visceral abscesses previously reported [2,3].

The pathogenesis of visceral abscesses often involves bacterial translocation from the gut or hematogenous spread, leading to localized suppuration. *A.* *hydrophila*, known for its production of aerolysin and enterotoxins, contributes to tissue destruction and an inflammatory response [4]. Complications of visceral abscesses can include septicemia, peritonitis, and multiorgan failure, necessitating prompt intervention [5]. Pancreatic abscesses caused by *A. hydrophila* are rare but potentially fatal, often progressing to sepsis and death.

In this case, the patient recovered following systemic antibiotic therapy with ceftriaxone and gentamicin. This case underscores the importance of considering rare and unusual microorganisms as potential causes of deep visceral abscesses. This report describes a 76-year-old female patient with a pancreatic tumor who presented with a synchronous abscess in the pancreatic region, where *A. hydrophila* was identified as the causative pathogen.

## Case presentation

We present the case of a 76-year-old female patient with no significant medical history who was admitted to the general surgery department for a suspected metastatic pancreatic neoplasm initially identified through imaging at another hospital. The patient reported a several-week history of epigastric pain, nausea, vomiting, and jaundice, with worsening symptoms over the past three days.

On examination, she appeared cachectic, with visible jaundice and general abdominal tenderness, mainly at the epigastric quadrant. Laboratory investigations showed elevated liver biomarkers: alanine aminotransferase (ALAT) at 185 U/L (normal: 10-40 U/L), aspartate aminotransferase (ASAT) at 200 U/L (normal: 10-40 U/L), lactate dehydrogenase (LDH) at 450 U/L (normal: 140-280 U/L), alkaline phosphatase (ALP) at 320 U/L (normal: 40-120 U/L), total bilirubin at 25.6 mg/dL (normal: 0.6-1.7 mg/dL), and direct bilirubin 13.2 mg/dL (normal: 0-0.3 mg/dL). C-reactive protein (CRP) was elevated at 75 mg/L (normal: <5 mg/L). A complete blood count revealed normal hemoglobin at 13.5 g/dL (normal: 13-17 g/dL), elevated white blood cells at 18,000/µL (normal: 4,000-11,000/µL), and normal platelets at 230,000/µL (normal: 150,000-450,000/µL). Renal function was within the normal range (Table 1).

**Table 1:** Laboratory results at admission.

Parameter	Value	Normal Range
Alanine aminotransferase (ALAT)	185 U/L	10-40 U/L
Aspartate aminotransferase (ASAT)	200 U/L	10-40 U/L
Lactate dehydrogenase (LDH)	450 U/L	140-280 U/L
Alkaline phosphatase (ALP)	320 U/L	40-120 U/L
Total bilirubin	25.6 mg/dL	0.6-1.7 mg/dL
Direct bilirubin	13.2 mg/dL	0-0.3 mg/dL
C-reactive protein (CRP)	75 mg/L	<5 mg/L
Hemoglobin	13.5 g/dL	13-17 g/dL
White blood cells (WBC)	18,000/µL	4,000-11,000/µL
Platelets	230,000/µL	150,000-450,000/µL
Creatinine (renal function)	0.9 mg/dL	0.6-1.2 mg/dL

**Figure 1:**
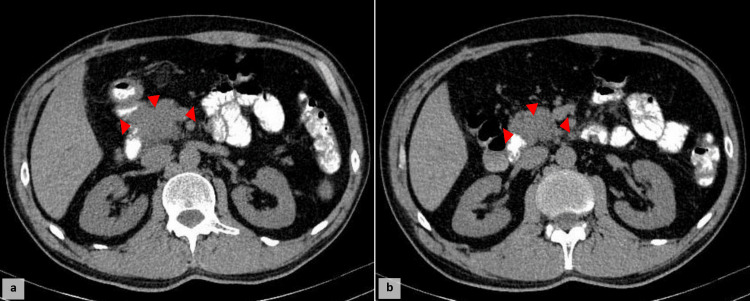
Axial CT scan showing (a) a lesion isodense to the pancreatic parenchyma (arrowhead) and (b) a poorly defined, hypodense lesion in the pancreatic head relative to the adjacent parenchyma during the pancreatic phase (arrowhead).

CT imaging revealed a 54×34×30 mm mass in the pancreatic head, causing marked dilation of the extrahepatic bile ducts (Figure 1). Multiple tissue-like lesions were noted in the lungs and parenchyma, suggestive of a metastatic disease.

**Figure 2:**
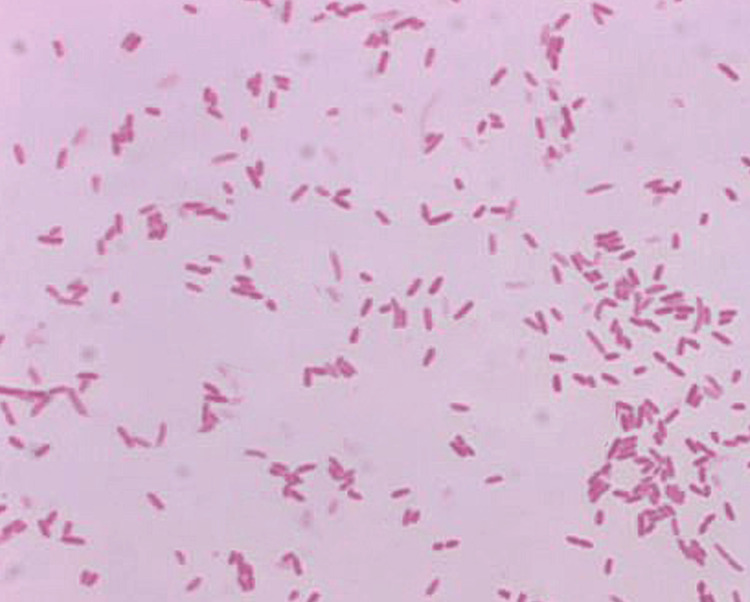
Gram-stained smear showing Gram-negative, rod-shaped bacteria characteristic of Aeromonas hydrophila (Gram stain, ×1000).

The patient underwent a choledochoduodenostomy to relieve bile duct obstruction. Intraoperatively, an abscess near the pancreatic mass was drained, and samples were sent for microbiological analysis. A biopsy of the pancreatic mass confirmed an adenocarcinoma of the pancreas. Microbiological analysis using Gram stain identified *A. hydrophila *as the causative organism (Figure 2).

The culture on blood agar displayed medium-sized, greyish, round, smooth, and convex colonies. The isolate was sensitive to ceftriaxone and gentamicin but resistant to cefoxitin and ampicillin (Table 2).

**Table 2:** Antibiotic resistance in isolated Aeromonas hydrophila.

Antibiotics	Resistance
Ceftriaxone	Sensitive
Ceftazidime	Resistant
Cefepime	Sensitive
Ertapenem	Resistant
Ciprofloxacin	Sensitive
Levofloxacin	Sensitive
Ofloxacin	Sensitive
Amikacin	Sensitive
Gentamicin	Sensitive
Tobramycin	Sensitive
Nitrofurantoin	Sensitive
Sulfamethoxazole - trimethoprim	Sensitive
Ampicillin	Resistant
Amoxicillin - clavulanic acid	Resistant
Piperacillin	Sensitive
Piperacillin - tazobactam	Sensitive
Cefadroxil (for cystitis)	Resistant
Cefuroxime	Intermediate
Cefoxitin	Resistant
Cefotaxime	Resistant

Intravenous ceftriaxone (2 g/day) and gentamicin (5 mg/kg/day) were initiated for synergistic coverage. The patient showed clinical improvement, with the fever and leukocytosis resolving by the fourth day of treatment.

## Discussion

*A. hydrophila* are halophilic, non-acid-fast rods [1], facultatively anaerobic bacilli, and Gram-negative microorganisms commonly found in soil, foodstuffs, and aquatic environments. In humans, *A. hydrophila* is most frequently associated with gastroenteritis, typically causing acute diarrhea often linked to foodborne intoxication [1,3]. However, in rare cases, it can lead to extra-intestinal infections such as sepsis or necrotizing fasciitis [4]. Other possible manifestations include abdominal abscesses, meningitis [5], and hepatobiliary infections [6]. Clinical symptoms may include tachypnea, tachycardia, hypotension, body temperature above 38°C, altered consciousness, and either leukocytosis or leukopenia [4].

Various microorganisms, including *Enterococcus*, *Escherichia*, *Pseudomonas*, *Klebsiella*, and *Enterobacter* species, are commonly isolated from bile. However, the incidence of *A. hydrophila* isolation remains low [7,8]. A review by Clark et al. highlighted the presentation of *Aeromonas* species in the bilio-pancreatic tract, primarily as cholangitis. Furthermore, the majority of *Aeromonas* infections occur in immunosuppressed patients or those with associated malignancies [2,9], as demonstrated in our case, since our patient did not have any history of immunosuppression. Disseminated infections are typically seen in individuals with underlying malignancies or chronic liver disease [6]. Although our case involves an incidental co-occurrence of a pancreatic neoplasm and *A. hydrophila* abscess, rather than a post-operative infection, rare reports have documented deep infections caused by *A. hydrophila*. In a different context, a cohort study by Ueno et al. found that the isolation rate of *A. hydrophila* in hepato-biliary-pancreatic (HBP) surgery was low, at approximately 0.75% of the 10,074 patients who underwent perioperative bacteriological examination. In some patients, this infection led to rapid clinical deterioration, potentially becoming life-threatening [4].

While deep visceral infections are rare and not commonly encountered in everyday clinical practice, the potential impact of *A. hydrophila* should not be underestimated [4,10]. Rare cases have demonstrated the involvement of *A. hydrophila* in deep abscesses, with reports of a pericholecystic abscess and a liver abscess [2,11]. Some strains of *A. hydrophila* can produce a toxin called aerolysin, a cytolytic substance that binds to the surface of eukaryotic cells and induces membrane perforation [12].

*Aeromonas* isolates are typically sensitive to aminoglycosides, trimethoprim/sulfamethoxazole, moxalactam, chloramphenicol, and tetracycline [6]. However, *A. hydrophila* often exhibits resistance to cefazolin, cefoxitin, and fosfomycin, which are commonly used for prophylaxis in HBP surgeries. Effective treatment options include broad-spectrum antibiotics such as third-generation cephalosporins, imipenem, or fluoroquinolones [4]. In our case, the combination of ceftriaxone and gentamicin was chosen to ensure broad-spectrum Gram-negative coverage while leveraging their synergistic effects against* A. hydrophila.*

## Conclusions

*A. hydrophila* is a rare but notable pathogen capable of causing deep visceral infections, including abscesses in the hepatobiliary and pancreatic regions. Despite its low incidence in clinical settings, its potential for causing severe conditions such as sepsis or necrotizing fasciitis should not be underestimated, particularly in immunocompromised patients or those with malignancies. Early identification through microbiological cultures and timely administration of appropriate antibiotics are crucial for effective treatment, as resistance to commonly used prophylactic antibiotics like cefoxitin and cefazolin is not uncommon. This case emphasizes the importance of considering *A. hydrophila *as a possible cause of deep visceral abscesses in patients with unexplained abdominal masses or metastatic disease.
